# A titratable murine model of progressive emphysema using tracheal porcine pancreatic elastase

**DOI:** 10.1038/s41598-023-41527-1

**Published:** 2023-09-14

**Authors:** Imani Joshi, Andrew J. Devine, Rashika Joshi, Noah J. Smith, Brian M. Varisco

**Affiliations:** 1 College of Arts and Sciences, Xavier University, Cincinnati, OH USA; 2 Cincinnati Children’s Hospital Medical Center, Cincinnati, OH USA; 3 University of Cincinnati College of Medicine, Cincinnati, OH USA; 4 University of Arkansas for Medical Sciences, 1 Children’s Way Slot 663, Little Rock, AR 72202 USA

**Keywords:** Chronic obstructive pulmonary disease, Preclinical research

## Abstract

Progressive emphysema often leads to end-stage lung disease. Most mouse models of emphysema are typically modest (i.e. cigarette smoke exposure), and changes over time are difficult to quantify. The tracheal porcine pancreatic elastase model (PPE) produces severe injury, but the literature is conflicted as to whether emphysema improves, is stable, or progresses over time. We hypothesized a threshold of injury below which repair would occur and above which emphysema would be stable or progress. We treated 8-week-old C57BL6 mixed sex mice with 0, 0.5, 2, or 4 activity units of PPE in 100 µL PBS and performed lung stereology at 21 and 84 days. There were no significant differences in weight gain or mouse health. Despite minimal emphysema at 21-days in the 0.5 units group (2.8 µm increased mean linear intercept, MLI), MLI increased by 4.6 µm between days 21 and 84 (p = 0.0007). In addition to larger MLI at 21 days in 2- and 4-unit groups, MLI increases from day 21 to 84 were 17.2 and 34 µm respectively (p = 0.002 and p = 0.0001). Total lung volume increased, and alveolar surface area decreased with time and injury severity. Contrary to our hypothesis, we found no evidence of alveolar repair over time. Airspace destruction was both progressive and accelerative. Future mechanistic studies in lung immunity, mechano-biology, senescence, and cell-specific changes may lead to novel therapies to slow or halt progressive emphysema in humans.

## Introduction

Progressive emphysema leads to end stage lung disease in many conditions like chronic obstructive pulmonary disease (COPD), alpha-1 antitrypsin deficient lung disease, Marfan syndrome, other connective tissue diseases, and other conditions^1–4^. Mouse models of progressive emphysema hold the potential to uncover key cellular and biological processes and lead to novel therapies to halt progressive alveolar enlargement.

Most models of progressive emphysema are either genetic or result from prolonged, chronic injury. Alpha-1 antitrypsin deficient emphysema has been modeled using both antisense oligonucleotide^5^ and genetic^6,7^ approaches, and both models demonstrate emphysema that worsens over time. The tight skin mouse has a single allele mutation of *Fibrillin-1* and develops spontaneous emphysema^8^. Many mice with mutations of other matrix and matrix-associated genes also develop spontaneous emphysema^9–12^. Emphysema worsens with long-term cigarette smoke exposure^13^ and after cessation of cigarette smoke exposure^14^. In general, genetic models produce more robust emphysema, while exposure models like cigarette smoke are less robust but thought to be more generalizable. While it is criticized as lacking clinical relevance, tracheal administration of porcine pancreatic elastase (PPE) or other proteases is one of the more commonly used exposure models.

Although tracheal administration of PPE is one of the more commonly used mouse models, whether emphysema is progressive in this model is controversial. Suki, et al. demonstrated worsened emphysema between days 2 and 21, following 6 activity units of tracheal PPE in B6 mice^15^, but a study from the same group found that there was no worsening and perhaps improvement in emphysema at days 2, 7, and 21 days with a dose of 0.25 units. Tracheal administration of 2.4 units PPE to B6 mice resulted in progressive emphysema between days 1 and 10 in B6 mice, but there was no progression of emphysema between days 10 and 21 in a study by Lucey, et al.^16^. Similarly, in hamsters, mean linear intercept (MLI) increased between days 1 and 5, but was stable between days 5 and 30 after 15 units to PPE^17^. In a rat study by Szabari, et al., there was no significant difference in alveolar diameter at 3, 21, and 105 days after tracheal PPE^18^. Our group has shown that by following mice up to 84 days, 2 units of PPE in B6 mice causes increased emphysema between days 21 and 84^7^, which is inconsistent with studies that showing stability and potentially alveolar repair with low to moderate doses of tracheal PPE.

We therefore hypothesized that there is a threshold of injury and alveolar airspace destruction that is determinative as to whether lungs undergo repair and regeneration or progressive airspace destruction. We tested this hypothesis using lung stereological measures in mice treated with differing doses of PPE at different time points.

## Materials and methods

All experiments were conducted in a manner consistent with the ARRIVE guidelines and in accordance with applicable policies and guidelines of Cincinnati Children’s Hospital Medical Center and the Cincinnati Children’s Research Foundation.

### Animal use and care

Animal use was approved by the Cincinnati Children’s Hospital IACUC (2020-0040). C57BL6 mice were purchased from Jackson Laboratories, housed in a barrier facility which provided filtered air, autoclaved chow, and purified water with 12-h light/dark cycles.

### Administration of tracheal PBS and PPE

Eight-week-old, 20–25 g male and female, mice were anesthetized with 2% isoflurane, tracheally cannulated with a 22-gauge angiocatheter with the needle tip broken off using a mouse intubating board, and administered 100 µL of PBS, 1 unit/mL PPE (Elastin Products Company, E134) in PBS, 4 unit/mL PPE, or 8 unit/mL PPE during inspiration by direct injection of the solution through the angiocatheter. The doses were not weight adjusted. The mice were recovered, and after ambulation resumed, returned to the colony.

### Animal sacrifice, lung harvest, and tissue processing

At 21 or 84 days after treatment, mice were anesthetized with intraperitoneal ketamine/xylazine/acepromazine (70/10/2 mg/kg) and exsanguinated by cutting the left renal artery and lungs flushed with 1 mL PBS via the right ventricle. Via a small neck incision, the trachea was cannulated with a 22-gauge blunt tip metal cannula, secured with 3–0 silk, and inflated with 4% paraformaldehyde (PFA) in PBS at 25 cm water pressure. This pressure was chosen to minimize the possibility of artifactual alveolar wall rupture. After inflation, the silk suture was secured, cannula was removed, and the lungs were removed from the chest. Lung volume was measured by water displacement by suspension in a beaker of water on a scale and fixed overnight in 4% PFA in PBS. After fixation, lung lobes were disarticulated and paraffinized. Paraffinized lobes were arranged with random orientations, embedded, and 5 µm sections were taken through all lobes.

### Mean linear intercept, alveolar thickness, and lung surface area determination

Sections were stained with hematoxylin and eosin. Mean Linear Intercepts (MLI) for each mouse were determined using the methods of Dunnill, et al.^19^ using a 10X objective lens and five fields from each lung lobe. For alveolar thickness (AWT) measures, each image was segmented into nine equal fields and two alveolar thickness measures were made in each field. The average MLI and alveolar thickness values per mouse were used in calculations. Alveolar surface area (SA) was calculated as lung volume divided MLI minus AWT.

$$SA= \frac{{V}_{Lung}}{MLI-AWT}$$

### Immunohistochemistry

Lung sections were immunostained for CD45-expressing cells using rabbit anti-mouse CD45 antibody (Cell Signaling #70257) at 1:1000 concentration using ABC Vectastain kit (Vector Laboratories PK-4000) per manufacturer protocol. Lung lobes were tile scanned at 4X magnification and high resolution on a NiE brightfield microscope (Nikon). Using Nikon elements analysis software, a binary object for lung tissue was defined as saturation above threshold and CD45-positive cells defined as intensity below threshold with a size limit of 5 to 20 square microns. The same analysis was applied to all images, and the number of CD45-positive cells per mm^2^ of lung tissue was calculated.

### Statistical analysis

Comparisons between groups were made using Welch’s t-test or ANOVA with Holm post hoc test for parametric data and the Mann–Whitney U-test or Kruskal–Wallis with Dunn’s post hoc test for non-parametric data using rstatix^20^ in the R statistical package. Data is presented as whisker plots with center bar representing mean and whiskers standard deviation for parametric data and box plots with center line representing median, box 25th and 75th percentile, and lines 2.5th and 97.5th percentiles. Dots represent individual data points and were created using ggpubr^21^. p-values less than 0.05 were considered statistically significant.

### Ethical approval

Animal use was approved by the Cincinnati Children’s Hospital IACUC (2020-0040).

## Results

### Mouse health

**Figure 1:**
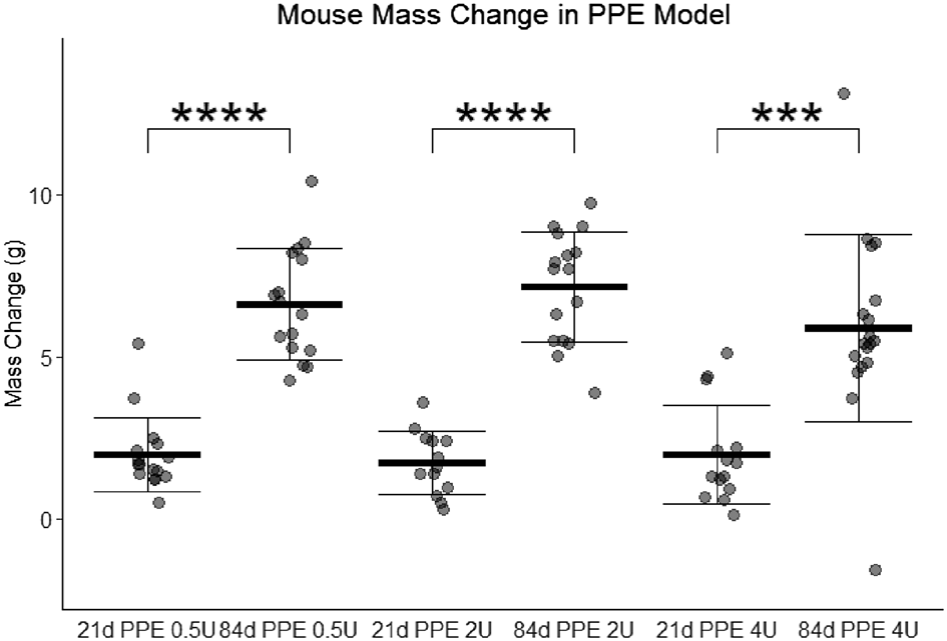
Mouse mass gain in the titratable PPE model. Between days zero and 21 and days 21 and 84, mice treated with 0.5, 2, or 4 activity units of porcine pancreatic elastase had similar increases in mass without significant differences between groups.

Mice were treated with PBS (zero activity units), 0.5 units, 2 units, or 4 units of tracheal PPE. No mice died in any of the experimental groups, and all mice gained weight over time with no significant differences between groups (Fig. 1). This finding allays concerns that difference in emphysema could be due to difference in overall mouse health.

### Airspace enlargement increases over time regardless of injury severity

**Figure 2:**
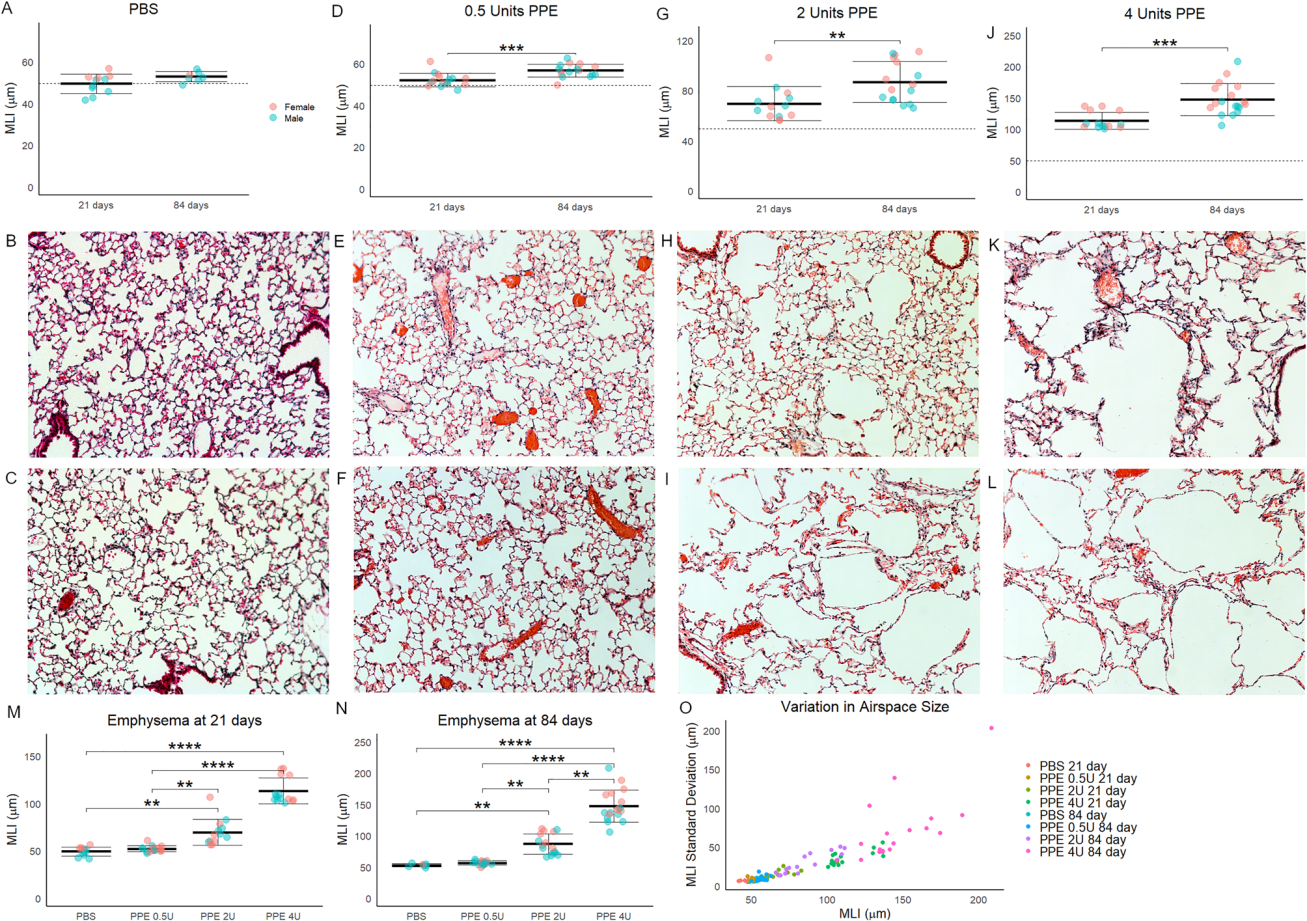
emphysema over time in the titratable PPE model. (**A**) Mice treated with only 500 µL of tracheal PBS (zero activity units of PPE) had a small but not significant increase in mean linear intercept (MLI) between days 21 and 84 post-administration. Dashed line represents 21 day PBS MLI. (**B**) A representative 10X photomicrograph from 21 and (**C**) 84 days. (**D**) While the MLI of mice treated with 0.5 units of PPE was slightly more than that of mice treated with PBS (dashed line), there was a small but significant increase in MLI at 84 days. (**E**) A representative 10X photomicrograph from 21 and (**F**) 84 days. (**G**) A larger increase in MLI was seen in mice treated with 2 units of PPE, and a larger increase over time was also seen. (**H**) A representative 10X photomicrograph from 21 and (**I**) 84 days. (**J**) Emphysema at 21 days and increase at 84 days was even greater after treatment with 4 units of PPE. (**K**) A representative 10X photomicrograph from 21 and (**L**) 84 days. (**M**) The increase in MLI at 21 days was not significant between PBS and 0.5 units of PPE but was significantly higher for 2 and 4 units. (**N**) These differences were more pronounced at 84 days. (**O**) Each mouse had 20 MLI measurements made of its lungs. The variability in these measures increased with greater emphysema. **p < 0.01, ***p < 0.001, ****p < 0.0001 by Welch’s t-test for 2-group and by holm post hoc test after ANOVA for 4-group comparisons.

These mice were sacrificed for lung and airspace evaluation at 21 and 84 days. Contrary to our hypothesis, mice treated with 0.5, 2, and 4 units of tracheal PPE all experienced progression of airspace simplification between days 21 and 84 (Fig. 2A-L). Notably however, there was no difference in MLI between PBS and PPE 0.5 unit-treated mice at 21 or 84 days (Fig. 2M,N) indicating that we could not differentiate injury-induced progressive emphysema from age-related airspace simplification. The variability in airspace size in different regions of the lung increased with increasing average MLI (Fig. 2O), but this variability was not lung lobe specific (Supplemental Fig. S1). Sex was not used as a variable in these analyses. Although in some groups female mice seemed to have larger MLI than male mice, none of these differences were statistically significant. These data show that regardless of injury severity, emphysema is progressive in the PPE model.

### Greater injury results in more rapid emphysema progression

**Figure 3:**
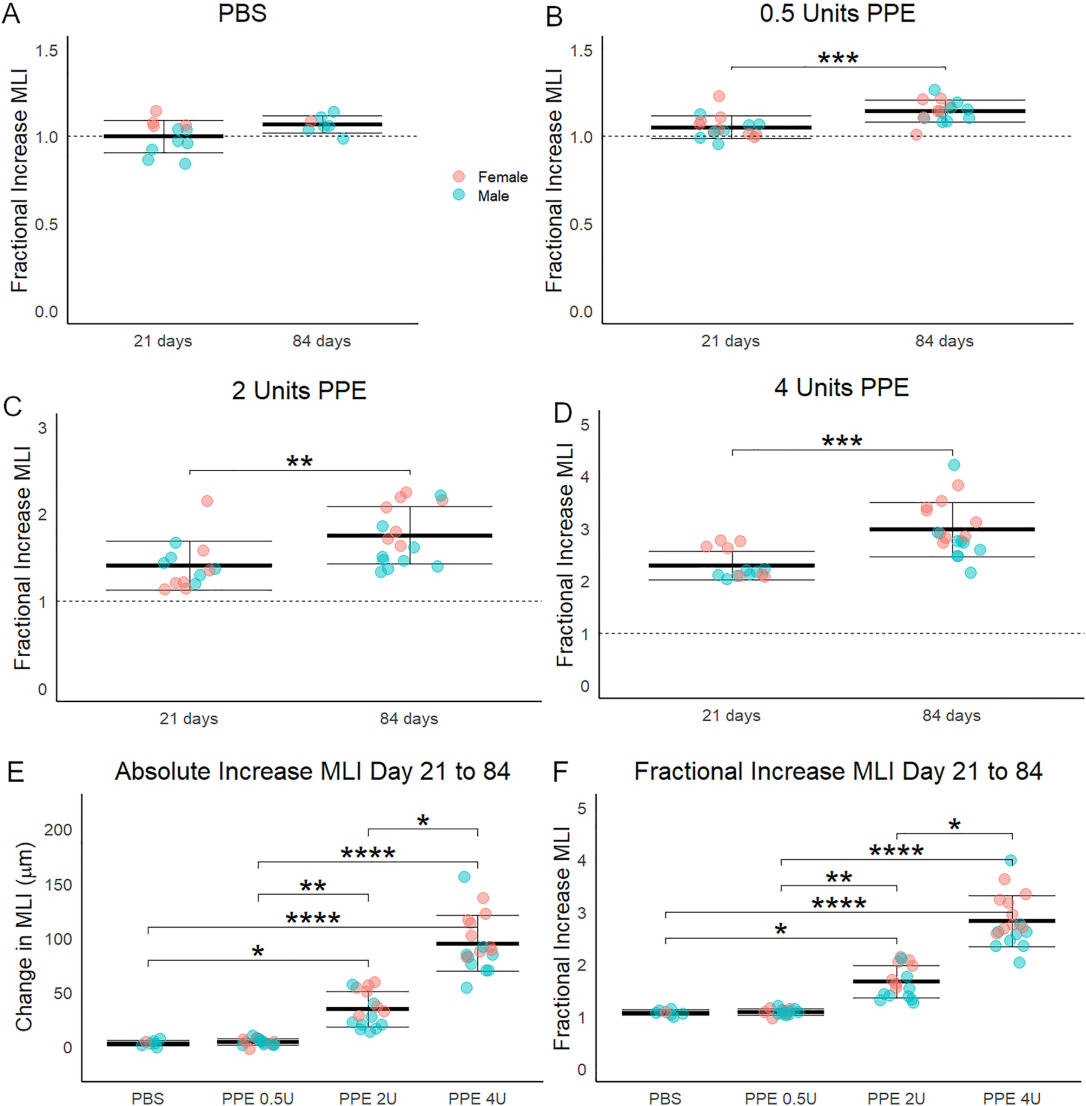
Emphysema acceleration in the titratable PPE model. (**A**) The fractional increase in mean linear intercept (MLI) between days zero and 21 and days 21 and 84 was not significant after PBS treatment, but (**B**) was significant after treatment with 0.5, (**C**) 2, and (**D**) 4 activity units of PPE. 21-day PBS treated MLI serves as reference for these calculations. (**E**) In comparing the absolute and (**F**) fractional increase of MLI between days zero and 21 and 21 and 84, there is a dose response in the absolute and relative increase in MLI. *p < 0.05, **p < 0.01, ***p < 0.001, ****p < 0.0001 by Welch’s t-test for 2-group and by holm post hoc test after ANOVA for 4-group comparisons.

To determine whether emphysema progressed at a constant or variable rate with different levels of injury, we assessed the relative increase in MLI over time. Alveolar simplification increased significantly in all PPE-treated, but not significantly in PBS-treated mice, and the relative increase appeared greater in mice treated with higher doses of PPE (Fig. 3A-D). In comparing the absolute (Fig. 3E) and relative (Fig. 3F) changes in airspace size between days 21 and 84, the lungs of mice treated with higher doses of PPE had more significant progression of emphysema compared to those treated with lower doses. We found no significant differences in MLI measurement by sex. These data show that in addition to more severe initial injury resulting in greater emphysema, emphysema progresses faster with greater initial injury.

### Total lung volume increase and alveolar surface area reduction are both progressive and more pronounced with worsening degrees of initial lung injury

**Figure 4:**
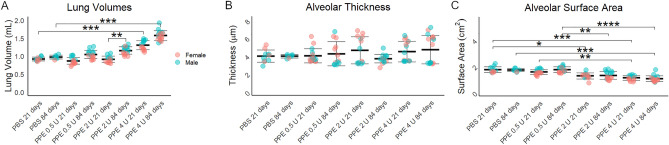
Lung volumes, alveolar septal thickness and alveolar surface area. (**A**) Lung volumes were greater in mice treated with higher doses of PPE, and between days 21 and 84, mice treated with 2 activity units of PPE had a significant increase in lung volume. (**B**) There were no differences in measured alveolar thicknesses. (**C**) Alveolar surface area was decreased to a greater degree in the lungs of mice treated with higher doses of PPE, but there were no significant differences in any group between day 21 and 84. *p < 0.05, **p < 0.01, ***p < 0.001, ****p < 0.0001 by holm post hoc test after ANOVA.

Increased severity of injury resulted in larger lung volume, and there was also an increase in lung volume between 21 and 84 days; although, this change was only significant in mice administered 2 units of PPE (Fig. 4A). Overall alveolar thickness was little different between groups (Fig. 4B). Alveolar surface area decreased more with more severe injury, but there were no significant differences between days 21 and 84 in any treatment group (Fig. 4C). These data indicate that the increased emphysema seen in this model is not due solely to tissue destruction but also airspace enlargement with resultant increased lung volume.

### Immune cells are increased following tracheal PPE

**Figure 5:**
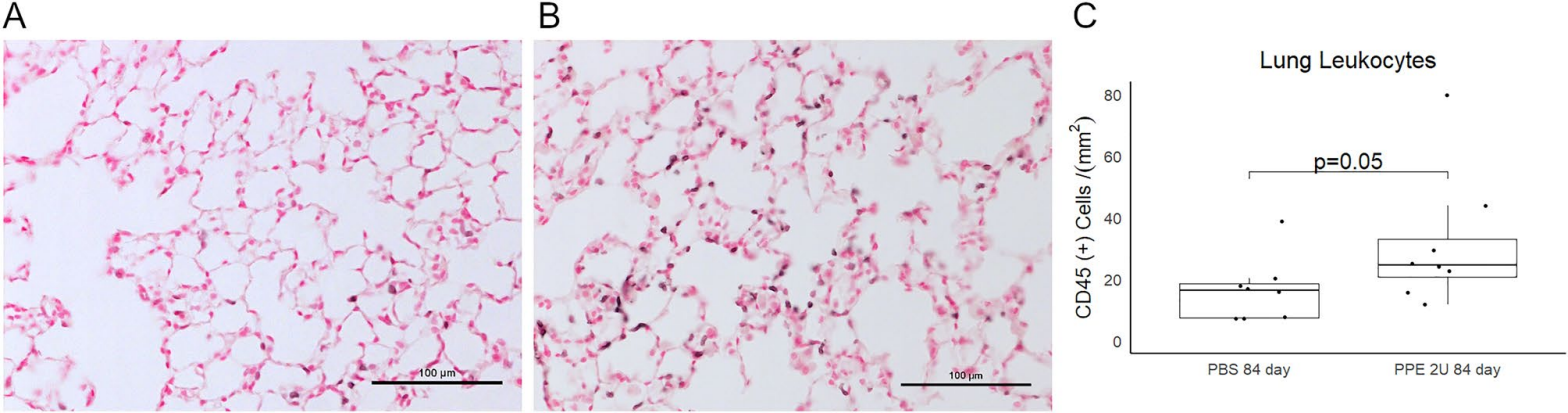
Inflammatory Cells in the Tracheal PPE Model. (**A**) The lungs of mice treated with either PBS or (**B**) 2 activity units (U) of tracheal porcine pancreatic elastase were evaluated for immune cells by immunostaining for CD45 and performing quantitative image analysis. Representative, higher magnification photomicrographs are shown. (**C**) Tile scans of lung lobes with quantification of immune cell number normalized to tissue area revealed a 50% increase in the number of CD45-positive cells. Comparison is by Mann–Whitney U-test.

We quantified the number of CD45-positive cells in the lung sections of mice treated with PBS or 2 units of PPE at 84 days. The median number of leukocytes was 50% higher in PPE-treated lung than PBS, and this difference neared statistical significance (Fig. 5). These data suggest that ongoing inflammation may be important in progressive emphysema.

## Discussion

This is the most comprehensive study to date with regards to characterizing the progression of emphysema in the murine tracheal PPE model of emphysema. Contrary to our hypothesis, we could not identify a threshold below which lung repair and regeneration would occur leading to repair of the damaged lung parenchyma. All doses of PPE tested resulted in progressive emphysema, and this progression was greater in lungs with the greater initial injury. It should be noted; however, that PBS-treated animals also had a non-significant increase consistent with age-related alveolar simplification playing a role in this model. Since in humans emphysema is often progressive despite medical optimization and elimination of injurious agents such as cigarette smoke^3,22–24^, this model has the potential to shed light upon underlying pathobiological processes.

Multiple potentially complimentary mechanisms could account for the finding of progressive emphysema over time. Among these are inflammation, altered protease-antiprotease balance, altered tissue mechanics, and senescence/premature aging. Inflammation and immune cell-mediated injury is perhaps the most widely studied potential driver of progressive emphysema^25^, and both innate and adaptive immune cells have been implicated. We found increased leukocytes in the lungs of PPE-treated mice. Neutrophils and macrophages have long been implicated in emphysema pathogenesis^26,27^. Both secrete a host of proteases that are thought to mediate direct effects and interact with other immune cells indirectly promoting emphysema. In addition to cell-autonomous roles in tissue destruction, there is likely a synergistic effect that matrix destruction itself has on macrophage recruitment and activation as the degradation products of collagen^28^ and other matrix proteins^29,30^ act as damage associated molecular patterns (DAMPs). There is likely a synergistic role of matrix destruction and inflammatory cell recruitment as both collagen^28^ and elastin^31^ degradation products recruit inflammatory cells to the lung. By characterizing the lung immune responses that are most important in emphysema progression versus initiation and leveraging available genetic tools to mechanistically test cell-specific roles, we can begin disentangling injury and response and develop targeted immune therapies to prevent emphysema progression.

The protease/antiprotease model holds that an imbalance between destructive lung proteases and neutralizing antiproteases are responsible for emphysema. It is the oldest model, and arose after the discovery of alpha-1 antitrypsin by Eriksson and Laurell in 1963^32^. *Neutrophil elastase*^33–35^, cathepsins^36^, *Chymotrypsin-like elastase 1*^5,7^ and matrix metalloproteinases^37–40^ have all be shown implicated in emphysema. While antiprotease restoration with purified alpha-1 antitrypsin is standard of care in deficient individuals^41^, different therapies inhibiting some of the proteases listed above are being developed^7,42–44^. *Alpha-1 antitrypsin*-deficient mice are particularly susceptible to tracheal PPE^7^. As many of the proteases important in emphysema development and progression are synthesized by immune cells, there are strong links between the protease/antiprotease and immune models.

The mechanosensitive model of emphysema progression predicts accelerative lung tissue destruction with increasing airspace size, as was seen in our model. The mammalian lung exists in equilibrium between the distending negative pressure of the pleural space and the inward elastic recoil of the lung parenchyma, and strain is distributed through alveolar walls, bronchi, and vascular structures. If alveolar walls are lost, additional strain is placed on adjacent ones predisposing those walls to failure^45^. Supporting the importance of this mechanism, increasing transpulmonary pressure by partial airway occlusion worsens emphysema following PPE^46^ and the binding of proteases to lung matrix components increases with lung strain^47,48^, linking this model with the protease-antiprotease one. Mathematical modeling of lung strain in human emphysema predicts bullae creation in upper lung lobes with striking accuracy^45^. Due to gravity, transpulmonary pressure is greater in upper lung regions compared to lower ones. Furthermore, lung protease, collagenase, and elastase activity increases with strain^49^. Longitudinal analysis of septal failure in the PPE model of emphysema demonstrated not only increased failure in septal walls placed under increased strain, but also increased associated inflammation^18^ suggesting synergy between mechanosensitive and immune-cell mediated mechanisms of emphysema progression. Studying the biomechanics of in vivo or ex vivo lung tissue is challenging but likely important since cyclic strain is both necessary for normal lung function and altered in emphysema.

The premature aging model of emphysema holds that cellular senescence and reduced numbers of progenitor cells leads to an acceleration of the normal alveolar simplification seen in aging. It is difficult to disentangle the effects of aging from experimental injury in in vivo longitudinal models of emphysema since alveolar simplification is a natural part of aging, and aging-related processes could be accelerated in these models. The concept of COPD and emphysema as a condition of premature aging is controversial^50^, but several studies support it as important in the gradual loss of alveolar architecture. Over time, the “transcriptomic noise” of all lung epithelial cell lineages increases as telomere lengths decrease^51^, perhaps contributing to observed changes in cell function^52^. Progenitor cell populations are reduced or capacity diminished with age^53,54^. The senescence-associated cell phenotype (SASP) is pro-inflammatory^55^ linking this model to previously noted ones, and genetic ablation of a key senescence pathway components protects against emphysema in both aging and after tracheal PPE instillation^56,57^. Human studies have linked polymorphisms in some of these genes with emphysema^58^. In other tissues, cyclic stretch induces cellular senescence by p53-dependent^59^ and p53-independent^60^ mechanisms potentially linking mechanosensitive and premature aging models; however in lung epithelial cells, cyclic strain is also a mitogen^61^ making extrapolation from other tissue findings challenging.

## Conclusions

In summary, the mechanisms underlying emphysema initiation and progression are complex, interconnected, and likely change over the course of the disease. In humans, this course often culminates in end stage lung disease lending urgency to understanding its natural history and to identifying key nodes of the network at different stages of the disease. By developing a simple, titratable, and relatively quick model of emphysema progression, some of the key elements that operate independently of a chronic stimulus like cigarette smoke can be elucidated and novel therapies to slow or halt emphysema progression in patients in whom it is already established can be developed.

### Supplementary Information

Supplementary Figure 1.

## Data Availability

All data used in the generation of this manuscript is available upon request to the corresponding author.

## Abbreviations

AWT: Alveolar wall thickness
COPD: Chronic obstructive pulmonary disease
MLI: Mean linear intercept
PFA: Paraformaldehyde
PPE: Porcine pancreatic elastase
U: Unit
