# Supplementary material for: A titratable murine model of progressive emphysema using tracheal porcine pancreatic elastase
Source: Sci Rep. 2023 Sep 14;13:15259. doi: 10.1038/s41598-023-41527-1 (PMC10502133; doi:10.1038/s41598-023-41527-1)
Supplement: Supplementary file 1 — Supplementary Figure 1. [file 41598_2023_41527_MOESM1_ESM.docx]

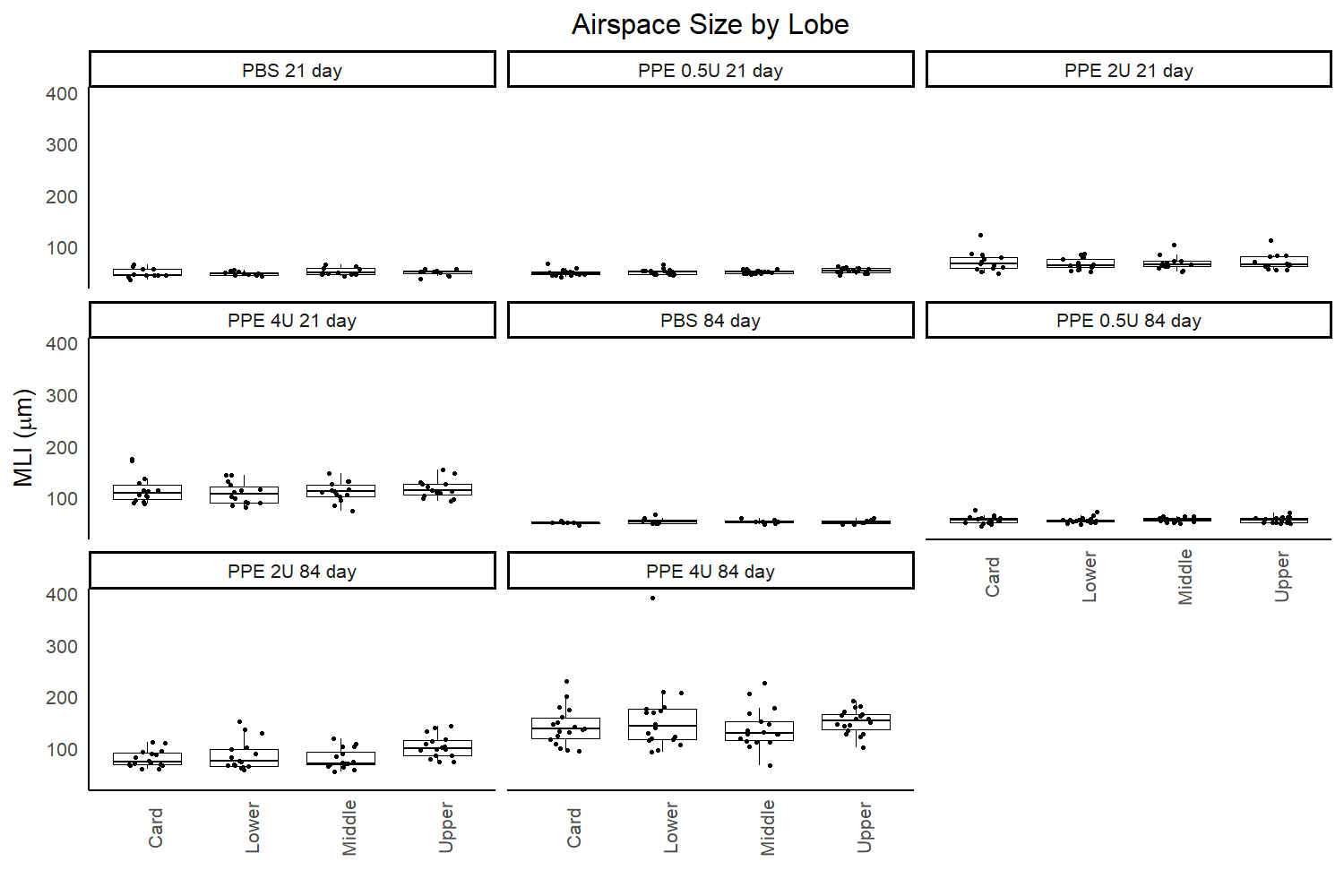


Supplemental Figure 1: Lobe-specific emphysema. While there was substantial variability of emphysema severity in different regions of the mouse lung, there was not a consistent lobar pattern to this emphysema.
